# Traumatic Brain Injury in an Elite Adolescent Soccer Goalkeeper: A Case Report

**DOI:** 10.7759/cureus.109088

**Published:** 2026-05-18

**Authors:** Adam Drummond, Hiren Patel, Richard Jackson, William Carlos

**Affiliations:** 1 Academy Medical Department, Burnley Football Club, Burnley, GBR; 2 Neurosurgery Department, Salford Royal NHS Foundation Trust, Salford, GBR

**Keywords:** concussion, elite athlete, football (soccer), head injury, soccer, sports-related concussion, traumatic brain injury

## Abstract

This case describes an 18-year-old English male professional soccer goalkeeper who sustained a significant head injury during training with transient loss of consciousness and seizure-like posturing exhibited on the field of play. Initial CT imaging in the emergency department demonstrated a focal intracranial haemorrhage within the septum pellucidum. Subsequent MRI identified multiple microhaemorrhages, predominantly within the right centrum semiovale, consistent with traumatic microhaemorrhages and possible underlying axonal injury. The player was admitted for hospital observation for four days. On discharge, he underwent an initial management period of prolonged rest for six weeks. A structured graduated return to sport programme was completed, incorporating serial neurocognitive assessment and repeat neuroimaging to guide progression. In the absence of definitive return to play guidance for structural brain injury, a conservative, clinically reasoned, specialist multidisciplinary team approach was adopted. The athlete returned to pre-injury competition level six months after injury without recurrence of symptoms. This case exemplifies the importance of distinguishing concussion from structural traumatic brain injury (TBI) and highlights considerations for safe return-to-play decision-making in the absence of evidence-based guidelines.

## Introduction

Soccer is associated with a substantial burden of head injury across all levels of participation. Large prospective injury surveillance studies in elite European soccer report overall injury rates of approximately eight injuries per 1,000 player-hours, with match play associated with significantly higher injury incidence than training [1]. Head and neck injuries represent a smaller but clinically important subset of these injuries, accounting for approximately 2-8% of all injuries in professional soccer, depending on study methodology and population [2]. Among these, concussion represents the most frequently reported neurological diagnosis, with reported incidence rates in elite soccer ranging from 0.06 to 0.14 concussions per 1,000 player-hours [3].

The 2022 Amsterdam Consensus Statement defines sports-related concussion (SRC) as a traumatic brain injury (TBI) induced by biomechanical forces, resulting in a rapid onset of short-lived neurological impairment that typically resolves spontaneously and is not associated with structural abnormalities on standard neuroimaging [4]. SRC is characterised by functional disturbance in the absence of structural abnormality detectable on conventional neuroimaging [4].

In contrast, structural brain injury following mild TBI (mTBI) is clinically important because it reflects visible tissue damage, rather than the primarily functional disturbance assumed in concussion, and is therefore a more severe injury. The presence of abnormalities on neuroimaging, such as traumatic microhaemorrhages, contusions, or diffuse axonal injury (DAI), indicates disruption of cerebral microvasculature and axonal structures, which is associated with a higher risk of prolonged symptoms or disability [5,6].

The true incidence of structural TBI within football is less clearly defined, as many head injuries are managed as a concussion without routine neuroimaging. There is growing evidence base regarding the long-term neurological consequences of repetitive head trauma, which has highlighted the risk of developing chronic traumatic encephalopathy (CTE), a progressive neurodegenerative condition associated with repeated concussive and subconcussive injuries [7]. Evidence from neuropathological and epidemiological studies demonstrates that cumulative head impacts can lead to abnormal tau protein deposition and neuroinflammation. These processes are associated with progressive cognitive, behavioural, and motor impairment, with clinical manifestations emerging years or decades after the initial injuries. This risk is particularly relevant in athletes involved in contact sports and exposed to repetitive brain trauma [8].

Recognition and management of head injury in elite soccer has evolved considerably in recent years. In response to growing concern regarding head injuries, governing bodies, including the Fédération Internationale de Football Association (FIFA) and the International Football Association Board (IFAB), have introduced concussion substitution trials, enhanced pitchside medical assessment protocols, and rule modifications aimed at improving player safety [9]. Further research and evidence-based refinement of clinical management strategies remain necessary to further mitigate the risk and long-term consequences of head injury in professional soccer.

In England, medical personnel working pitchside in professional soccer are mandated to have appropriate pre-hospital medicine training, which is delivered by the Football Association in the form of the Advanced Medical Management in Football (ATMMiF) course. The ATMMiF course provides structured training in pitchside trauma management, including airway management, spinal immobilisation, catastrophic injury management, and concussion recognition [10].

Pitchside and office-based assessment tools, such as the Sport Concussion Assessment Tool 6 (SCAT6), are designed to detect suspected SRC acutely but are not sufficient to exclude structural brain injury [4]. In cases involving significant mechanisms of injury, persistent symptoms, loss of consciousness, seizure activity, or other red flag features, urgent neuroimaging is required to identify intracranial pathology [11]. The presence of structural brain injury is associated with a higher risk of prolonged symptoms and disability in comparison to SRC [5].

This case report highlights diagnostic considerations for structural brain injury in elite sport, including the role of neuroimaging. It aims to illustrate the implications for clinical decision-making and the current uncertainty surrounding safe return-to-play strategies in athletes with structural TBI, particularly in the absence of evidence-based guidelines.

## Case presentation

A previously healthy 18-year-old professional male soccer goalkeeper, with no significant past medical history, sustained a head injury during a training match in January 2025. The injury occurred following a high-velocity collision, with a teammate's knee impacting the right temporal region of his head. Immediately following the collision, the player was observed to be lying motionless, a concerning 'observable sign', which, as per SCAT6 on-field assessment recommendations, requires immediate removal from play [4].

On approach, the player was confirmed to have a normal pulse and breathing rate, but was unresponsive to verbal and pain stimuli. Prompt airway assessment revealed obstructive sounds suggestive of partial airway compromise. Therefore, in the absence of signs suggestive of basal skull fracture, an appropriate nasopharyngeal airway was inserted to maintain airway patency. During airway management, the athlete developed brief tonic posturing of the upper limbs lasting approximately 10 seconds, a further SCAT6 observable sign [4]. High-flow oxygen at 15 L per minute was administered via a non-rebreather mask. After approximately one minute, the player's level of consciousness began to improve. He was awake with spontaneous eye opening and able to follow commands, but remained confused and disoriented to time, place, and person. A focused examination revealed no cervical spine tenderness or cranial deformity. Secondary assessment revealed no other distracting injuries. Neurological examination of the upper and lower limbs demonstrated normal power, sensation, and reflexes. In view of the mechanism of injury and red flag symptoms, full spinal 'triple' immobilisation was implemented using a cervical collar, head blocks, and straps prior to transport via ambulance to the nearest emergency department for further evaluation.

Computerised tomography (CT) performed in the emergency department demonstrated a 6 mm focus of increased attenuation on the right side of the cavum septum pellucidum, just superior to the thalami. The radiological appearance was suggestive of acute blood. To exclude atypical cyst pathology, follow-up neuroimaging was recommended to confirm the nature of the lesion. Magnetic resonance imaging (MRI) performed the following day demonstrated multiple small microhaemorrhages in both cerebral hemispheres, predominantly within the right centrum semiovale, identified on susceptibility-weighted imaging. Initial hospital radiological interpretation suggested these lesions may represent chronic post-traumatic changes from previous head injury.

The player remained hospitalised for four days. During this period, he experienced symptoms of headache, jaw pain, and fatigue. No focal neurological deficits were observed during admission. At nine days post injury, the player reported complete resolution of symptoms and was able to undertake activities of daily living without symptom exacerbation. SCAT6 assessment compared to baseline preseason testing was normal.

Following discharge, a specialist sports neurosurgical review was sought with subsequent multidisciplinary (MDT) neuroradiology discussion. Incorporating the acute clinical presentation and the presence of a new focal hyperdensity on CT, absent on prior comparison imaging, these findings were considered consistent with an acute haemorrhagic process. MRI demonstrated susceptibility-weighted signal changes in keeping with microhaemorrhages.

In the context of recent trauma, the MDT consensus was that these findings most likely represented acute traumatic microhaemorrhages. While the distribution did not involve classical regions associated with moderate-to-severe DAI, such patterns are less well defined in milder injury. The appearances were therefore considered compatible with traumatic microhaemorrhages, with a probable underlying mechanism of axonal shear injury.

At this stage, the MDT team was convened to guide return to sport planning, including the team sports medicine physician, neurosurgeon, physiotherapists, and sports rehabilitation staff. Due to the presence of structural abnormalities on imaging, the injury was managed as a traumatic brain injury rather than an uncomplicated concussion.

Acute phase: 0-6 weeks

Prolonged cognitive and physical rest was prescribed for a period of six weeks, followed by repeat MRI neuroimaging and neurocognitive testing. An initial six-week period of relative rest and symptom-limited activity was implemented to allow resolution of the acute metabolic and inflammatory responses initiated by the injurious process. This also reduced the risk of repeat injury.

Neurocognitive testing was performed using the Sport Concussion Office Assessment Tool (SCOAT6) and the Immediate Post-Concussion Assessment and Cognitive Testing (ImPACT, version 5.0) [4,12]. ImPACT was used under licence (Licence number: CYDVXSRKOM). Retesting at six weeks post injury demonstrated no ongoing symptoms, and cognitive scores were comparable to the athlete's preseason baseline testing. Repeat MRI, as demonstrated in Figure 1, continued to demonstrate multiple microhaemorrhages within the right centrum semiovale and septum pellucidum, consistent with possible DAI. Encouragingly, there was no increase in haemorrhage and no evidence of cerebral volume loss.

**Figure 1:**
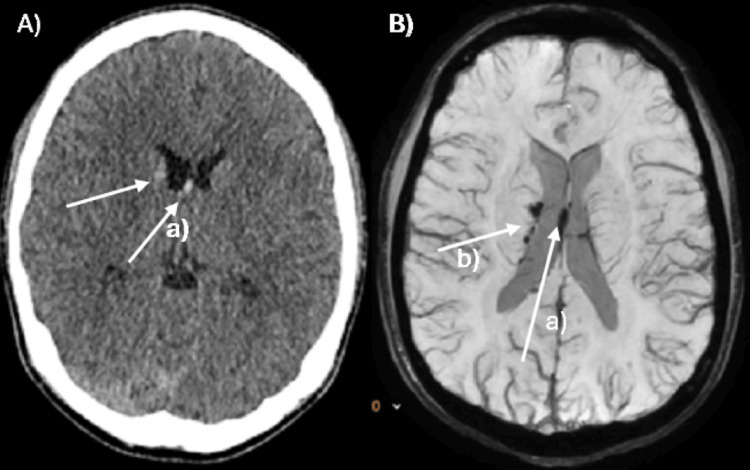
Neuroimaging at acute presentation. Initial CT (A) and six-week follow-up susceptibility-weighted MRI (SWI) (B) demonstrating intracranial haemorrhagic abnormalities and cerebral microhaemorrhages. (A) Axial CT head at presentation showing focal hyperdensity within the septum pellucidum and periventricular region (arrows), in keeping with acute haemorrhage. (B) Corresponding SWI sequence demonstrating well-defined, hypointense (black) foci (arrows) in the areas corresponding to the hyperdensity (white focus) seen on the CT head. SWI is highly sensitive to paramagnetic blood products (e.g., haemosiderin, ferritin, deoxyhaemoglobin), supporting the presence of haemorrhagic injury. Findings are suggestive of traumatic microhaemorrhages in the context of acute head injury.

Early conditioning: 6-12 weeks

A graduated return to activity programme was initiated over the following six weeks, broadly aligned with established concussion return-to-play principles, but applied more conservatively given the presence of structural brain injury. The primary aims during this phase were progressive reintroduction of physical activity, restoration of cardiovascular conditioning, and minimisation of risk for incidental head impact.

Rehabilitation commenced with low-intensity indoor conditioning, initially via static cycling at approximately 50% of maximum heart rate, with gradual progression in intensity as tolerated. Isometric neck strengthening exercises were introduced during this phase, informed by observational data in professional rugby union demonstrating that greater isometric neck strength is associated with a reduced incidence of concussion; specifically, a 10% increase in strength has been associated with an approximate 13% reduction in risk, although a direct causal relationship remains unproven [13].

Gym-based rehabilitation progressed to include strength and plyometric exercises, with return to running initiated at approximately eight weeks post injury. This was followed by the introduction of position-specific movement patterns without ball involvement. Throughout this phase, diving, contact activities, and ball work were avoided.

All training was conducted individually under one-to-one medical supervision, with ongoing symptom monitoring to ensure no recurrence or exacerbation of symptoms.

Sports-specific reintegration: 12-24 weeks

At approximately 12 weeks post injury, sport-specific rehabilitation was initiated, beginning with non-contact goalkeeper drills aimed at restoring neuromuscular performance, coordination, and reaction speed. Concurrently, gym-based training focus shifted from plyometric activities to the development of strength and power to support the physical demands of their goalkeeper position.

On-field activities were progressed in a graded manner, initially incorporating ball-handling drills without diving. Controlled diving was subsequently introduced, followed by increasing exposure to more dynamic and unpredictable movements. As tolerance improved, the athlete was gradually reintegrated into modified team training, with contact restricted initially through structured drills designed to minimise collision risk.

In parallel, the athlete resumed participation in team meetings and tactical analysis sessions to challenge cognitive processing and dual-task performance. Training intensity and complexity were progressively increased, culminating in full goalkeeper training during the final three weeks of rehabilitation.

Figure 2 demonstrates an overview of the staged rehabilitation process the athlete completed. The athlete returned to unrestricted team training and competitive play approximately six months following the initial injury. 

**Figure 2:**
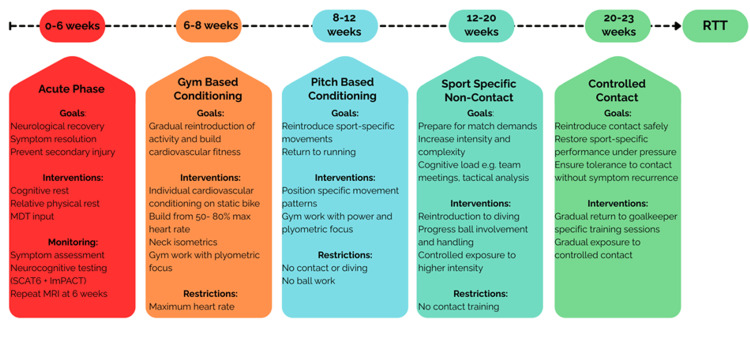
Schematic overview of the staged rehabilitation and return-to-training (RTT) pathway, illustrating the progressive phases from initial rest through conditioning, sport-specific reintegration, and return to full team training. This is an original figure created by the authors using Canva (Canva Pty Ltd., Perth, Australia); no external sources or copyrighted materials were used. Neurocognitive assessment tools incorporated within the rehabilitation pathway include the Sport Concussion Assessment Tool 6 (SCAT6) and the Immediate Post-Concussion Assessment and Cognitive Testing (ImPACT, version 5.0) [4,12].

## Discussion

TBI in sports

TBI in sports represents a spectrum ranging from mild concussion to severe structural brain injury involving intracranial haemorrhage, contusion, or DAI [4]. In elite soccer, the majority of reported head injuries are classified as SRC; however, structural brain injuries may occasionally occur, particularly following high-energy collisions involving rapid rotational acceleration-deceleration forces that produce shear strain across white matter tracts [14].

DAI is a common pathological consequence of rotational head trauma. Rapid acceleration-deceleration forces generate shear strain across white matter tracts, particularly within vulnerable regions such as the corpus callosum, brainstem, and centrum semiovale. These shearing forces disrupt axonal membranes, resulting in impaired neuronal connectivity and microvascular injury. The presence of cerebral microhaemorrhages on susceptibility-weighted MRI sequences is widely recognised as a radiological marker of traumatic axonal injury [5, 15].

In this case, the distribution of microhaemorrhages within the right centrum semiovale and septum pellucidum was anatomically consistent and suggestive of DAI. Importantly, structural abnormalities on neuroimaging alter patient risk modelling. Athletes with prior significant head injury demonstrate increased susceptibility to further injury and potentially prolonged recovery trajectories [6].

Neuroimaging recommendations in sporting head injury

CT remains the first-line imaging modality for acute head injury due to its ability to rapidly detect life-threatening intracranial haemorrhage. Current National Institute for Health and Care Excellence (NICE) guidelines recommend urgent CT imaging in patients presenting with features such as loss of consciousness, post-traumatic seizure, Glasgow Coma Scale score below 15, focal neurological deficit, or signs of skull fracture [11].

MRI provides greater sensitivity for detecting parenchymal injury, particularly non-haemorrhagic axonal injury and small cerebral microbleeds. SWI sequences are especially useful for identifying microhaemorrhages that may not be visible on CT. Consequently, MRI plays an important complementary role in evaluating patients with persistent neurological symptoms or suspected traumatic axonal injury following head trauma [15]. Microbleeds visible on SWI sequences are increasingly recognised in moderate-to-severe TBI and are associated with shearing forces affecting deep white matter structures such as the centrum semiovale [5].

In elite sport settings, access to advanced neuroimaging and specialist neuroradiology review may facilitate more accurate diagnosis of structural mTBI and guide management decisions.

Player recovery

Recovery following TBI involves a complex neurometabolic cascade characterised by ionic imbalance, excitatory neurotransmitter release, mitochondrial dysfunction, impaired glucose metabolism, and reductions in cerebral blood flow. These processes create a temporary energy crisis within neurons, rendering the brain more vulnerable to secondary physiological stressors [16].

In addition, traumatic microhaemorrhage exposes surrounding brain tissue to blood degradation products such as heme and iron, which trigger a local inflammatory response involving microglial activation, astrocyte activation, and cytokine release. While the acute inflammatory response typically evolves over days to weeks, residual low-grade glial activation may persist for longer periods. Although the precise duration of these pathophysiological processes is difficult to define, current evidence suggests that the major metabolic disturbances and early inflammatory responses generally resolve over several weeks, while subtle neuroinflammatory changes may persist for longer. Importantly, biological recovery does not always parallel clinical symptom resolution, and the absence of symptoms does not necessarily imply complete physiological recovery [17].

These considerations influenced the initial conservative management approach used in this case. The athlete demonstrated rapid symptom resolution within approximately nine days; however, a repeat MRI at six weeks demonstrated persistent structural abnormalities consistent with traumatic microhaemorrhages and possible axonal injury. This discordance between clinical recovery and radiological findings highlights the limitations of symptom-based return to play decisions.

Given the presence of structural abnormalities on imaging suggestive of mTBI and therefore proposed greater pathophysiological insult, a more cautious rehabilitation timeline was considered appropriate. While imaging findings do not directly quantify injury severity, they may reflect ongoing neurobiological processes beyond clinical symptom resolution. Accordingly, a conservative recovery approach was adopted, recognising that this strategy is based on clinical reasoning in the context of limited direct evidence rather than definitive guidance.

The initial rehabilitation strategy therefore prioritised six weeks of prolonged relative rest, followed by slow, gradual reintroduction of physical activity, and careful monitoring for symptom recurrence. Progressive conditioning was undertaken in a controlled environment to minimise the risk of further head impact while allowing restoration of cardiovascular fitness and sport-specific skills. This was followed by a graduated, stepwise return to sport programme, more consistent with established concussion return-to-play principles.

Guidelines on return to play

Current return-to-play protocols in soccer are primarily designed for the management of SRC. While consensus guidelines provide structured graduated return to sport pathways for concussion in soccer, equivalent evidence-based protocols for athletes with structural TBI are lacking [18]. In the United Kingdom's professional soccer leagues, including the Premier League, there are currently no published guidelines outlining when it is safe for a soccer player to return to sport following structural brain injury.

The absence of clear evidence-based guidelines in soccer and wider sport highlights the need for further research into the long-term neurological outcomes and safe return to play timelines for athletes sustaining structural brain injuries in sports.

In cases of structural mTBI, there is limited high-quality evidence to guide safe reintroduction to exercise and sport. In cases demonstrating evidence of structural TBI, return-to-play decisions must therefore be individualised and guided by MDT clinical judgement. Factors to consider include the severity and location of structural injury, time frame of symptom resolution, neurocognitive recovery, and stability of neuroimaging findings. Return-to-sport clearance should also account for the risk of re-injury, and therefore the risk of developing long-term neurological sequelae, such as CTE.

Due to the limited high-quality evidence defining a safe interval for return to collision sport following structural brain injury, the timing of return-to-contact sport in this case was considered in the context of the broader TBI literature. Observational research examining non-neurosurgical elective procedures after TBI suggests that additional physiological stressors, including general anaesthesia, may be associated with poorer neurological outcomes when performed early in the recovery period. Consequently, some authors have recommended delaying elective non-neurosurgical procedures for up to six months following recovery from TBI, particularly in cases involving structural injury, reflecting the prolonged period during which neurobiological and metabolic recovery may continue [19,20].

In the absence of definitive return-to-play guidance for athletes with structural brain injury, the rehabilitation timeline adopted in this case sought to balance the athlete's functional recovery with the theoretical risk of exacerbating ongoing neurobiological processes that may persist beyond the resolution of clinical symptoms. This approach was informed in part by literature examining recovery trajectories following TBI in other clinical contexts; however, it is acknowledged that such comparisons are indirect and may not be directly applicable to athletic return-to-play decision-making. As such, this strategy should be considered hypothesis-driven and based on clinical reasoning in the context of limited sport-specific evidence, rather than representing an evidence-based standard.

Neurocognitive testing is widely used in the assessment and management of SRC. SCAT6/SCOAT6 and ImPACT are examples of neurocognitive assessment tools [4,12]. Such testing evaluates domains including memory, attention, processing speed, and symptom burden. Baseline preseason athlete testing enables comparison with post-injury results and can assist clinicians in determining cognitive recovery. While these tools are validated for concussion assessments, they are not currently validated for structural TBI, and their utility remains less clearly defined. Normalisation of neurocognitive test results does not necessarily indicate complete neurological recovery, especially in the presence of persistent structural abnormalities on imaging. For this reason, neurocognitive testing was interpreted alongside clinical assessment and neuroimaging findings.

The athlete returned to competitive play six months after injury without recurrence of symptoms or neurological deficit. During this time, the athlete safely returned to their usual educational activities and driving. The athlete requires no further specialist neurosurgical follow-up.

## Conclusions

This case report highlights the challenges of return-to-play decision-making in athletes with structural brain injury in the absence of evidence-based guidelines. A comprehensive approach incorporating repeat neuroimaging, neurocognitive testing, and multidisciplinary clinical evaluation was used to guide return-to-sport decisions. Further research is required to clarify the clinical significance of neuroimaging abnormalities in athletes and to support the development of evidence-based return-to-play guidelines for athletes with structural TBI.
